# Supplementary material for: Inhibition of Human Neutrophil Functions In Vitro by Multiple Sclerosis Disease-Modifying Therapies
Source: J Clin Med. 2020 Nov 2;9(11):3542. doi: 10.3390/jcm9113542 (PMC7692529; doi:10.3390/jcm9113542)
Supplement: Supplementary file 1 [file jcm-09-03542-s001.pdf]

Supplemental Table 1. Effect of DMTs on HS PMN intracellular killing activity of *K. pneumoniae.*

Time (min)	Controls	First-line treatment		Second-line treatment		Statistical analysis
		INFβ-1b	GA	NAT	FTY	Mann–Whitney test
	Mean Survival Index ± SEM **(% of initial microbial population killed by PMNs)**					
	A	B	C	D	E	
30	1.51±0.02 (49%)	1.87±0.01 (13%)	1.77±0.06 (23%)	1.69±0.05 (31%)	>2 (0%)	*P* = 0.0003 A *vs.* B<br>*P* = 0.0010 A *vs.* C<br>*P* = 0.003 A *vs.* D<br>*P* < 0.0001 A *vs.* E
60	1.74±0.04 (26%)	>2 (0%)	1.94±0.04 (6%)	1.96±0.05 (4%)	>2 (0%)	*P* = 0.0003 A *vs.* B<br>*P* = 0.04 A *vs.* C<br>*P* = 0.004 A *vs.* D<br>*P* < 0.0001 A *vs.* E
90	>2 (0%)	>2 (0%)	>2 (0%)	>2 (0%)	>2 (0%)	n.s.

$P < 0.05$ was deemed statistically significant

Abbreviations: DMTs, disease-modifying therapies; HS, healthy subject; PMNs, polymorphonuclear leukocytes; IFNβ-1b, interferonβ-1b; GA, glatiramer acetate; NAT, natalizumab; FTY, fingolimod

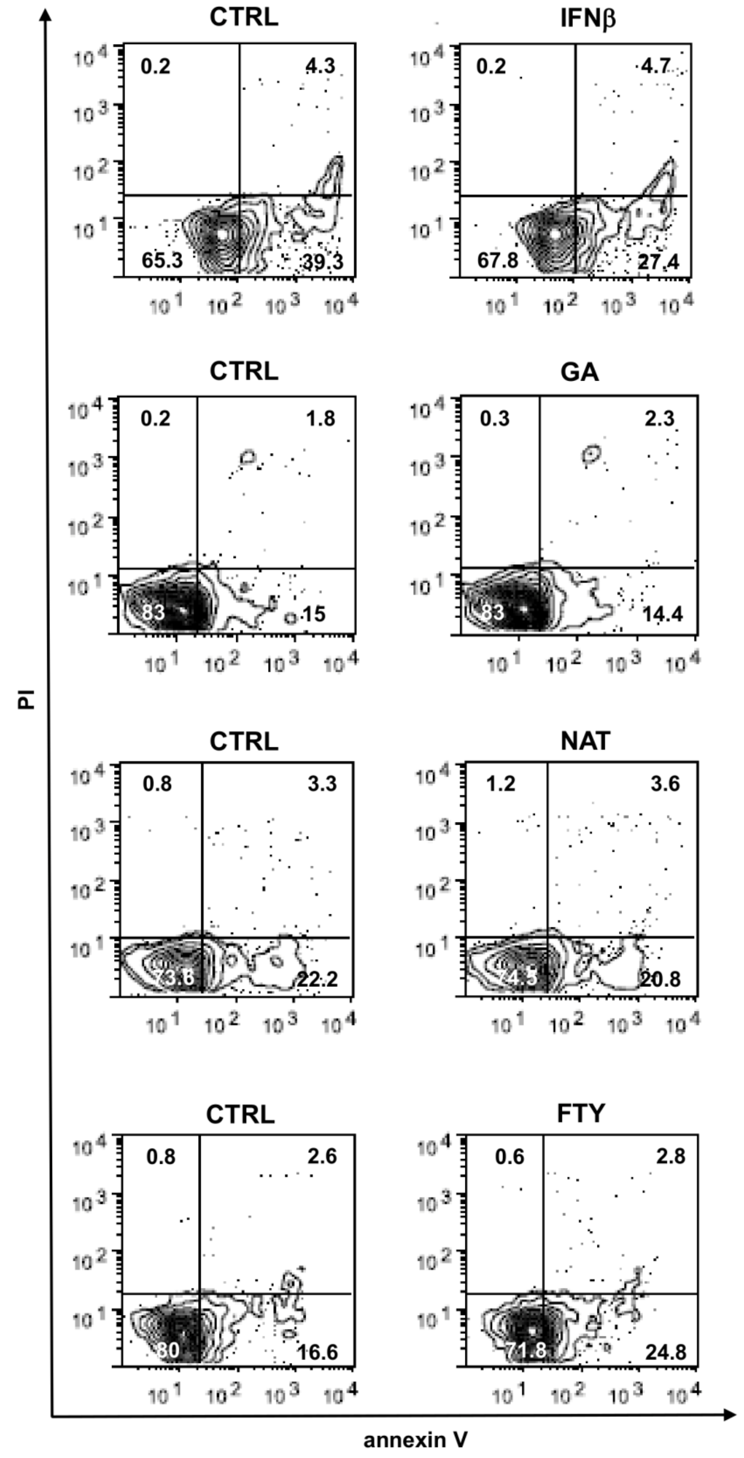

Supplemental Figure 1. Effect of DMTs on HS PMN apoptosis. Representative dot plots of untreated (left panels) or DMTs treated PMNs (right panels). Viable cells are presented in the lower left quadrant, early apoptotic cells in the lower right quadrant, late apoptotic cells in the upper right quadrant and unviable cells in the upper left quadrant.

© 2020 by the authors. Licensee MDPI, Basel, Switzerland. This article is an open access article distributed under the terms and conditions of the Creative Commons Attribution (CC BY) license (http://creativecommons.org/licenses/by/4.0/).
